# Role of CXCR4 in the progression and therapy of acute leukaemia

**DOI:** 10.1111/cpr.13076

**Published:** 2021-05-29

**Authors:** Long Su, Zheng Hu, Yong‐Guang Yang

**Affiliations:** ^1^ Key Laboratory of Organ Regeneration & Transplantation of the Ministry of Education The First Hospital Jilin University Changchun China; ^2^ National‐Local Joint Engineering Laboratory of Animal Models for Human Diseases Changchun China; ^3^ International Center of Future Science Jilin University Changchun China; ^4^ Department of Hematology The First Hospital Jilin University Changchun China

**Keywords:** CXCR4, acute myeloid leukaemia, acute lymphoblastic leukaemia, haematopoietic stem cell transplantation

## Abstract

CXCR4 is expressed on leukaemia cells and haematopoietic stem cells (HSCs), and its ligand stromal‐derived factor 1 (SDF‐1) is produced abundantly by stromal cells in the bone marrow (BM). The SDF‐1/CXCR4 axis plays important roles in homing to and retention in the protective BM microenvironment of malignant leukaemia cells and normal HSCs. CXCR4 expression is regulated by multiple mechanisms and the level of CXCR4 expression on leukaemia cells has prognostic indications in patients with acute leukaemia. CXCR4 antagonists can mobilize leukaemia cells from BM to circulation, which render them effectively eradicated by chemotherapeutic agents, small molecular inhibitors or hypomethylating agents. Therefore, such combinational therapies have been tested in clinical trials. However, new evidence emerged that drug‐resistant leukaemia cells were not affected by CXCR4 antagonists, and the migration of certain leukaemia cells to the leukaemia niche was independent of SDF‐1/CXCR4 axis. In this review, we summarize the role of CXCR4 in progression and treatment of acute leukaemia, with a focus on the potential of CXCR4 as a therapeutic target for acute leukaemia. We also discuss the potential value of using CXCR4 antagonists as chemosensitizer for conditioning regimens and immunosensitizer for graft‐vs‐leukaemia effects of allogeneic haematopoietic stem cell transplantation.

## Graphical abstract

CXCR4 is expressed on leukaemia cells and its ligand stromal‐derived factor 1 (SDF‐1) is produced by stromal cells in the bone marrow. The SDF‐1/CXCR4 axis plays an important role in homing to and retention in bone marrow of leukaemia cells. During the last three decades, CXCR4 has been studied as a potential target for treatment of acute leukaemia.

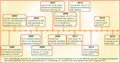

## 1 INTRODUCTION

Chemokines are small peptides with molecular weights of 8‐12 KDa, which are secreted by multiple types of cells, such as immune cells, stromal cells and tumour cells. Chemokine receptors are seven‐transmembrane G‐protein‐coupled receptors (GPCRs), and one receptor can bind to multiple chemokines.^1^, ^2^ Conversely, one chemokine can recognize several receptors.^1^, ^2^ CXCR4 was first discovered as a cofactor facilitating the entry of human immunodeficiency virus (HIV) into CD4^+^ T cells and was then classified into GPCR subfamily.^3^, ^4^ CXCR4 is widely expressed in many types of cells, including haematopoietic stem cells (HSCs), T lymphocytes, B lymphocytes, monocytes, macrophages, epithelial cells, endothelial cells and neurons.^5^ Stromal‐derived factor 1 (SDF‐1), also known as CXCL12, is the only ligand for CXCR4 but can also bind to CXCR7. After the engagement of SDF‐1 and CXCR4, many intracellular pathways are activated, including RAS‐MAPK, PI3K‐AKT‐mTOR and JAK‐STAT, which then regulate chemotaxis, gene expression and cell survival.^6^, ^7^ *Sdf1* or *Cxcr4* homozygous mutations in mice resulted in embryonic lethality, and the development of B lymphocytes and myeloid cells was severely impaired.^8^, ^9^ Other defects, including cardiac ventricular septal defect and defective formation of large vessels supplying the gastrointestinal tracts, were found.^10^ HSCs express high levels of CXCR4 and can migrate from the foetal liver to the bone marrow (BM) along with the SDF‐1 gradient participating in the transformation of haematopoietic sites in different stages of individual development.^11^ After birth, SDF‐1 secreted by stromal cells recruits HSCs into the BM niche to regulate quiescence or proliferation.^12^ CXCR4 contributes to lung alveolar regeneration after pneumonectomy.^13^ SDF‐1 expression is upregulated after tissue injury, promoting the migration of CXCR4^+^ adult stem cells to injury lesions to protect or repair infarcted cardiac and ischaemic cerebral tissues.^14^, ^15^ The treatment of ischaemic diseases by mobilized tissue‐committed stem cells was reviewed by Kwon *et al*.^16^ CXCR4 is a co‐receptor for the entry of HIV type 1 (HIV‐1) into CD4^+^ T cells, which was prevented by SDF‐1.^4^, ^17^ Therefore, increasing efforts have been made to develop new CXCR4 antagonists to control HIV infection (reviewed by Zhang *et al*).^18^ CXCR4 also plays important roles in the development, invasion, angiogenesis, epithelial–mesenchymal transition and maintenance of stemness of tumour cells,^19^, ^20^, ^21^, ^22^, ^23^ and targeting CXCR4 is a potential therapeutic strategy for treating malignant tumours.^24^, ^25^, ^26^

Acute leukaemia (AL) includes acute myeloid leukaemia (AML) and acute lymphoblastic leukaemia (ALL). AML is the most common AL in adult patients, while ALL is the first and second most frequent AL in children and adults, respectively.^27^, ^28^ Except for acute promyelocytic leukaemia (APL), chemotherapy remains the backbone of treatment for other AL subtypes. In recent years, with the use of tyrosine kinase inhibitors and chimeric antigen receptor T cells in AL, patients’ survival has increased to some extent. However, there is still a considerable scope for improving patients’ outcomes.^27^, ^29^, ^30^, ^31^ Haematopoietic stem cell transplantation (HSCT) is the regular treatment for patients with AL, including autologous HSCT (auto‐HSCT) and allogeneic HSCT (allo‐HSCT). The graft‐vs‐leukaemia (GVL) effects of allo‐HSCT mediated by allogeneic T cells can effectively eradicate residual leukaemia cells. However, relapse remains a major obstacle for successful treatment. Therefore, more effective treatment methods are needed to eliminate residual leukaemia cells after allo‐HSCT.

## 2 ROLE OF CXCR4 IN AML

### 2.1 CXCR4 participates in homing and residence of AML cells in BM

CXCR4 was critical for murine BM engraftment by human severe combined immunodeficient repopulating stem cells.^32^ Human cells pretreated with CXCR4 antibodies impeded engraftment and in vitro CXCR4‐dependent migration to SDF‐1 of CD34^+^CD38^‐/low^ cells associated with in vivo engraftment and stem cell function.^32^ CXCR4 expression influences the engraftment of autologous stem cells in patients undergoing auto‐HSCT. Significantly faster haematologic recovery was found in patients who received transplanted CD34^+^ cells that showed high spontaneous and SDF‐1‐induced migration.^33^ Therefore, SDF‐1/CXCR4 plays a critical role in homing to and retention in the BM of normal HSCs.

Like normal HSCs, CXCR4 is also closely associated with the migration of AML cells.^34^ Higher SDF‐1‐induced migration was observed in AML for CD34^+^ BM‐derived cells than in paired CD34^+^ peripheral blood (PB)‐derived cells, and a lower percentage of circulating leukaemia blasts in patients with a relatively high level of SDF‐1 induced migration indicated the role of CXCR4 in the anchoring of leukaemia cells in the BM.^34^ In 2004, Monaco *et al* evaluated the engraftment of AML cells into NOD/SCID mice.^35^ Six of the 11 patient samples were engrafted successfully. Poor prognosis was observed to be inversely correlated with engraftment, and the median overall survival (OS) was 26.1 weeks for patients with cell engraftment and 95.9 weeks for those without. No correlation between CXCR4 expression and engraftment was found, and anti‐CXCR4 antibody failed to block the engraftment of AML cells.^35^ Concurrently, CXCR4‐dependent engraftment of AML cells into NOD/SCID mice has been reported.^36^ Although AML cells from some patients did not express cell surface CXCR4, intracellular CXCR4 expression was detected in all samples. Pretreatment of human AML cells with neutralizing CXCR4 antibodies blocked their homing to the BM and spleen of NOD/SCID/β2M^null^ mice and treating mice previously engrafted with AML cells with antibodies against CXCR4 resulted in a dramatic decrease in leukaemia cell levels in a dose‐ and time‐dependent manner.^36^ Subsequently, a debate on whether engraftment of AML cells into mouse BM was dependent on SDF‐1/CXCR4 between these two groups was published.^37^ The opposite observations may be associated with different mice used and if newly expressed CXCR4 was inhibited.^37^

Recently, a murine MLL‐AF9‐driven AML model was used to evaluate the engraftment of leukaemia cells into mouse BM.^38^ The deletion of *cxcr4* in AML cells eradicated leukaemia cells in vivo, but their homing to the BM was not impaired. Furthermore, SDF‐1 is dispensable for the development of leukaemia in mice. Thus, CXCR4 signalling may play an essential role in AML stem cells, preventing differentiation independent of SDF‐1.^38^ Using high‐resolution 2‐photon and confocal intravital microscopy of mouse calvarium BM, chemoresistant MLL‐AF9 AML cells were found to become less motile and unaffected by AMD3100.^39^ Therefore, there may be other factors that regulate the homing and retention of AML cells within the BM. However, whether such phenomena possess leukaemia‐type specificity remains unclear.

### 2.2 CXCR4 expression and its regulation in AML

AML cells exposed to low oxygen partial pressure showed upregulated expression of CXCR4, and the underlying mechanisms involved alteration of lipid rafts.^40^ *NPM1* is one of the most common mutated genes in AML, and increased CXCR4 expression was observed when NIH3T3 cells were transfected with plasmids encoding *NPM1* mutation A with enhanced migration and invasion abilities.^41^ AML blasts with mutated *NPM1* displayed significantly higher CXCR4 expression than those without.^42^ However, no significant correlation between *NPM1* mutation and CXCR4 or phosphorylated CXCR4 (pCXCR4) expression was observed in the BM specimens of untreated AML patients.^43^ *CEBPA* mutations consist of unilateral and bilateral mutations, whereas only bilateral mutations indicate a favourable prognosis. N‐terminal *CEBPA* mutations may impair CXCR4 expression, as only CEBPA p42 can recognize the CXCR4 promoter by chromatin immunoprecipitation assays.^44^ *FLT3*‐ITD mutation is an indicator of poor prognosis for patients with AML and associates with upregulated CXCR4 expression in a series of studies.^45^, ^46^, ^47^ The downstream pathways may involve STAT5 and Pim‐1.^45^ Epigenetic regulation of CXCR4 expression by miR‐146a has been reported in patients with different subtypes of AML.^48^ Chemotherapy‐induced upregulation of CXCR4 expression was observed in both AML cell lines and clinical samples, which may represent a mechanism of treatment‐induced resistance in AML.^49^ Accordingly, the expression of CXCR4 in AML is regulated by multiple mechanisms, indicating a complicated role of CXCR4.

### 2.3 Relationship between CXCR4 expression and prognosis of AML

The unfavourable prognostic indication of CXCR4 expression in AML has been well documented in many studies.^42^, ^46^, ^47^, ^50^, ^51^, ^52^, ^53^, ^54^ AML patients with <20% CXCR4^+^/CD34^+^ cells had significantly superior OS and relapse‐free survival (RFS) than those with ≥20%.^46^ In a prospective study, patients with AML were divided into groups with low, intermediate or high levels of CXCR4 expression, as determined by CXCR4 mean fluorescence intensity ratio thresholds of <5, 5‐10 and ≥10, respectively, which resulted in significantly different outcomes.^50^ AML patients with normal karyotype showed higher percentages of CXCR4^+^ cases than those without, and high CXCR4 expression predicted poor prognoses in multivariate analysis.^52^ A combination of CXCR4 and VLA‐4 expression can divide AML patients into different groups with various prognoses.^53^ In paediatric patients with AML, high CXCR4 expression indicated an unfavourable prognosis only in the low‐risk group.^54^ Taken together, CXCR4 expression levels show prognostic indications in AML and may be a potential marker for re‐stratifying the prognosis of patients with AML.

### 2.4 Targeting CXCR4 in treatment of AML

#### 2.4.1 *CXCR4* *small molecular antagonist AMD3100/AMD3465*

The first generation of CXCR4 antagonist AMD3100 inhibited the migration of AML blasts induced by SDF‐1 and their proliferation in vitro and reversed the enhanced engraftment of AML blasts into NOD/SCID mice mediated by SDF‐1.^55^ Tavor *et al* found that AMD3100 could significantly inhibit proliferation and induce apoptosis in multiple AML cell lines^56^ and upregulate the expression of CD15 and CD11b.^56^ AMD3465 is the second generation of CXCR4 antagonist that can inhibit the migration of AML cells induced by SDF‐1 and multiple intracellular signalling pathways responsible for cell survival.^57^ AMD3465 partially reversed the protective effects of stromal cells on leukaemia cells in vitro. AMD3465 alone or combined with granulocyte colony‐stimulating factor (G‐CSF) mobilize leukaemia cells from the BM and render them killed by chemotherapeutic drugs or sorafenib in leukaemic mice, leading to reduced leukaemia burden and prolonged survival.^57^ In a similar study of a murine APL model, AMD3100 also reversed the drug resistance of AML cells mediated by stromal cells in vitro and reduced leukaemia burden and prolonged survival of leukaemic mice when used with chemotherapy.^58^

Cocultivation of *FLT3*‐ITD mutated AML blasts or haematopoietic progenitor cells (HPCs) on BM stromal cells resulted in a strong proliferation advantage compared with *FLT3*‐wide‐type AML blasts, and addition of AMD3100 to the co‐culture significantly reduced the proliferation of *FLT3*‐ITD mutated cells, but did not affect *FLT3*‐wide‐type cells.^59^ AMD3100 promoted the death of leukaemia cells with high CXCR4 expression and reduced NOG leukaemia‐initiating cells but had no efficacy when AML cells did not express CXCR4.^60^ This suggests that CXCR4 expression levels may be a potential marker for identifying candidates who can benefit from CXCR4 antagonists. A triple combinational therapy using AMD3100 and anti‐PD‐L1 plus chemotherapy was investigated in a mouse AML model. Noticeable benefits of triple combinational therapy could be achieved to eradicate leukaemia blasts that transformed into prolonged survival of mice. The frequencies of regulatory T cells (Tregs) and myeloid‐derived suppressor cells in the PB of mice treated with triple combinational therapy consistently decreased.^61^ Collectively, conventional chemotherapeutic drugs, kinase inhibitors or immune checkpoint inhibitors are potential strategies to be combined with CXCR4 antagonists to enhance the eradication of AML.

In 2009, the first case report of using AMD3100 in a relapsed patient with AML who underwent sibling donor allo‐HSCT was reported.^62^ A significant decrease in leukaemia cell levels was observed after the patient was treated with AMD3100 plus chemotherapy, and a second allo‐HSCT was performed thereafter. Complete remission (CR) was achieved one month after transplantation. Five months after allo‐HSCT, the patient died of severe graft‐vs‐host disease (GVHD), but maintained continuous CR.^62^ Three years later, the first clinical trial of combination therapy with AMD3100 and chemotherapy in 52 patients with relapsed or refractory AML was reported.^63^ AMD3100 was increased to a maximum of 240 μg/kg/d without any dose‐limiting toxicities. An overall CR and CR with incomplete blood count recovery (CRi) rate of 46% were achieved in 46 patients treated with AMD3100 plus chemotherapy. Furthermore, no evidence of symptomatic hyperleukocytosis or delayed haematopoietic cell recovery was found.^63^ The efficacy and safety of chemotherapy combined with AMD3100 and G‐CSF in the treatment of relapsed or refractory AML were evaluated by the same group.^64^ No dose‐limiting toxicities were observed when AMD3100 was increased to a maximum of 750 μg/kg/d. However, this clinical trial was terminated early due to unsatisfactory responses after interim analysis.^64^ The POE 10‐03 trial was released in 2017 by the paediatric oncology experimental therapeutics investigators’ consortium.^65^ Nineteen patients were enrolled, including 13 with AML, 5 with ALL and 1 with myelodysplastic syndromes (MDS). AMD3100 was administered for 5 days at four dose levels (6, 9, 12 and 15 mg/m^2^/dose daily) followed by high‐dose cytarabine (every 12 hours) and etoposide (daily) 4 hours later. No dose‐limiting toxicities were found, and febrile neutropenia and hypokalaemia were the most common grade 3 or higher non‐haematologic toxicities attributable to AMD3100. Mobilization of leukaemia blasts into the PB was observed in 14 of 16 evaluable patients. All three patients achieved CR/CRi with AML.^65^ In phase I/II study of AMD3100 in combination with fludarabine, idarubicin, cytarabine and G‐CSF (FLAG‐Ida) for the treatment of patients with early‐relapsed or refractory AML, the CR/CRi rate was 50% among primary refractory and 47% among early‐relapsed patients, and three patients died during induction.^66^ Thus, AMD3100 plus FLAG‐Ida resulted in a relatively high CR/CRi rate in adult patients with primary refractory or early‐relapsed AML with acceptable toxicity. AMD3100 combined with the hypomethylating agent decitabine was used to treat newly diagnosed elderly patients with AML in phase I clinical trial (n = 69), with an overall response of 43%, and the most common side effects were myelosuppression and infection.^67^

#### 2.4.2 *New* *peptide or antibody antagonists of CXCR4*

New antagonists of CXCR4 in preclinical and clinical studies are summarized in Table 1. These antagonists not only inhibit SDF‐1 or stromal cell‐induced chemotaxis of leukaemia cells, but also impair the proliferation or induce death of leukaemia cells directly. Thus, when used alone or in combinational therapies, CXCR4 antagonists were found to significantly inhibit the growth of leukaemia cells and prolong the survival of leukaemic mice. It is worth noting that LY2510924 and PF‐06747143 have entered phase I clinical trials. Although some of these antagonists were suggested to be more potent than AMD3100, further preclinical and clinical studies are needed to confirm it.

**TABLE 1:** New peptide or antibody antagonists of CXCR4 in preclinical or clinical studies

Drugs	Types	Functions in vivo or in vitro
RCP168^68^	Peptide	Inhibit SDF‐1 or stromal cell‐induced chemotaxis of leukaemia cells
		Block the binding of 12G5 to cell surface CXCR4
		Induce apoptosis in stroma‐cocultured AML cells harbouring FLT3 mutation
E5^69^, ^70^, ^71^	Peptide	Inhibit SDF‐1 or stromal cell‐induced chemotaxis of leukaemia cells
		Induce concentration‐dependent apoptosis in AML cell lines
		Inhibit growth of HL‐60 cells in vivo and prolong survival of leukaemic mice
		Micelle formulation of E5 is a promising therapeutic approach for AML
LY2510924^72^, ^73^, ^74^	Peptide	Inhibit SDF‐1‐induced chemotaxis and prosurvival signals of AML cells
		Chiefly inhibit the proliferation of AML cells with little induction of cell death
		Mobilize the BM leukaemia cells into PB
		Anti‐leukaemia effects as monotherapy or in combination with chemotherapy
		Enhance the efficacy of quizartinib against *FLT3*‐ITD mutated AML cells
		Phase 1 trial: 4/11 patients achieved CR treated with LY2510924 plus chemotherapy (NCT02652871); dose escalation to a higher dose will be planned
PF‐06747143^75^, ^76^	Antibody	Inhibit SDF‐1‐induced chemotaxis of leukaemia cells
		Induce leukaemia cell death through its Fc‐effector function
		Inhibit growth of leukaemia cells in vivo and prolong survival of leukaemic mice
		Phase 1 trial is terminated due to a change in sponsor prioritization (NCT02954653)
BL‐8040^77^	Peptide	Mobilize the BM leukaemia cells into PB
		Induce differentiation of AML cells
		Induce apoptosis of AML cells in vivo and in vitro
		Synergize with BCL‐2 inhibitors or FLT3 inhibitors
HC4319^78^, ^79^	Peptide	Inhibit SDF‐1 and stromal cell‐induced chemotaxis of leukaemia cells
		Reverse drug resistance mediated by stromal cells
		Inhibit growth of U937 cells in vivo and prolong survival of leukaemic mice

#### 2.4.3 *Other* *strategies that target CXCR4*

Ibrutinib, an inhibitor of Bruton's tyrosine kinase (BTK), is used to treat Waldenström's macroglobulinaemia, mantle cell lymphoma and lymphoblastic leukaemia, which also inhibits SDF‐1 induced AKT and MAPK activation, leading to the inhibition of the migration and proliferation of leukaemia cells.^80^ Downregulation of CXCR4 expression by small interfering RNA (siRNA) is a potential strategy to treat many diseases, including AML. Lipopolymer/siRNA complexes are used to decrease CXCR4 expression, resulting in the inhibition of AML cell proliferation and chemosensitization.^81^ Dual‐function polycation (PCX)/siRNA nanoparticles can simultaneously inhibit CXCR4 expression and deliver siRNAs that target key oncogenes in AML cells.^82^ Monomethyl auristatin E conjugated with the CXCR4‐targeted protein nanoparticles could be utilized to kill CXCR4^+^ AML cells and to reduce leukaemia burden in mice without the severe toxicity of classical AML therapeutic drugs.^83^

#### 2.4.4 *CXCR4* *is potential target for immunotherapy*

The frequencies of Tregs in PB and BM of AML patients were higher than those in healthy controls. Increased CXCR4 expression robustly promoted the migration of Tregs towards BM, which played critical roles in immunosuppression of conventional T cells through proliferation inhibition, apoptosis promotion and suppression of IFN‐γ production.^84^ Using a murine MLL‐AF9 AML model, blocking CXCR4 was found to reduce Treg accumulation in the leukaemia haematopoietic microenvironment and promote anti‐leukaemic effects of CD8^+^ T cells, and delay leukaemia progression.^85^

#### 2.4.5 *CXCR4* *and differentiation syndrome*

Differentiation syndrome is a common complication of APL. Differentiated APL cells expressed high levels of CXCR4, and SDF‐1 secreted by lung cells could help these cells migrate to lung tissues, which was reduced by pretreatment with an anti‐CXCR4 antibody. Therefore, targeting CXCR4 may provide the basis for potential prophylaxis or treatment of differentiation syndrome.^86^

## 3 ROLE OF CXCR4 IN ALL

### 3.1 CXCR4 in the pathogenesis of ALL

The precursor B‐cell line Nalm‐6 selectively localized within the BM stroma, which was partially controlled by the SDF‐1/CXCR4 axis in vitro.^87^ Patient B‐ALL cells express high levels of CXCR4, and SDF‐1 stimulation can induce strong calcium fluxes and increased transendothelial migration.^88^ CXCR4 antagonists inhibited the chemotaxis and migration of B‐ALL cell lines and leukaemia blasts to BM stroma.^89^ Nalm‐6 cells pretreated with SDF‐1 showed reduced CXCR4 expression and homing to BM by 72 ± 16%, and leukaemia cell engraftment was significantly reduced (22 ± 11% vs 48 ± 5%).^90^ Murine BM contains unique anatomic regions defined by a specialized endothelium that expresses the adhesion molecule E‐selectin and SDF‐1 in discrete, discontinuous areas. It is CXCR4 blockade, not the loss of E‐selectin, that severely impedes homing of Nalm‐6 cells to these vascular niches.^91^ Similarly, CXCR4 is crucial for the homing and retaining of T‐ALL cells in the BM and stemness of T‐ALL.^92^, ^93^ ALL with MLL gene rearrangements (MLL +ALL) has a dismal outcome due to its insensitivity to chemotherapy. MLL +ALL cells expressed both CXCR4 and CXCR7, but chemotherapeutic agent‐induced apoptosis of leukaemia cells was inhibited by pretreatment with a CXCR4 inhibitor and accelerated by pretreatment with a CXCR7 inhibitor.^94^ Furthermore, patient B‐ALL cells or Nalm‐6 cells pretreated with SDF‐1 showed a doubling of adhesion to fibronectin, laminin and VCAM‐1.^90^ Collectively, these results indicate that SDF‐1/CXCR4 regulates the migration and chemosensitivity of ALL cells and their homing to BM and enhances the interaction between leukaemia cells and the extracellular matrix.

Although migration of B‐ALL and human CD34^+^ cells increased towards SDF‐1 concentrations, a significant decrease in migration towards very high SDF‐1 levels was only observed in B‐ALL cells.^95^ This difference may be due to the distribution of intracellular and cell surface CXCR4 between normal and malignant human HPCs.^95^ Moreover, VLA‐4 and Rho proteins are critical for B‐ALL cell homing to BM, but not for normal CD34^+^ cells.^95^ In a study using 27 clinical samples, leukaemia cells from all the patients showed SDF‐1‐dependent proliferation, but some did not undergo chemotaxis in response to SDF‐1 due to the absence of phosphorylation of p38 MAPK.^96^ However, loss of the chemotactic response of ALL cells to SDF‐1 did not impede their engraftment in NOD/SCID mice.^96^ SDF‐1‐mediated signalling through p38 MAPK is required for the homing of ALL cells, but not for normal PB CD34^+^ cells.^97^ Therefore, the different chemotactic responses and signalling pathways of normal CD34^+^ and ALL cells may shed light on their therapeutic implications.

B‐cell precursor ALL (BCP‐ALL) cells migrated significantly more towards ALL +mesenchymal stem cell (MSC) co‐cultures than towards MSC mono‐cultures, and such preferential migration of BCP‐ALL cells towards the leukaemia niche was not affected by AMD3100. Furthermore, there was no significant difference in SDF‐1 levels in these two culture systems’ supernatants.^98^ These results suggest that other factors regulate the migration and homing of leukaemia cells after the leukaemia niche is created.

### 3.2 CXCR4 expression and its regulation in ALL

Most studies indicated that ALL cell lines and primary ALL blasts expressed high CXCR4 levels,^92^, ^94^, ^99^, ^100^ while heterogeneity of CXCR4 expression in ALL was also reported in 100 paediatric patients with relapsed BCP‐ALL.^101^ Inactivation of Rac1 significantly prolonged the chemotactic response of ALL cells to SDF‐1, and this effect was associated with an alteration of CXCR4 internalization.^102^ CXCR4 expression was reduced in calcineurin‐deficient T‐ALL cells due to downregulation of cortactin expression, impinges CXCR4 trafficking.^93^ Inhibitors of histone deacetylases extensively downregulated CXCR4 expression at both mRNA and protein levels in leukaemia cell lines and lymphoblasts from patients.^103^ Accordingly, CXCR4 expression in ALL may show heterogeneity.

### 3.3 Role of CXCR4 in extramedullary invasion of ALL cells

Levels of CXCR4 expression determined by flow cytometry in lymphoblasts were associated with extramedullary organ infiltration (EOI) in childhood ALL (n = 73). EOI was defined as ultrasonographically measured enlargement of the liver or spleen. The fluorescence intensity of CXCR4 in leukaemia cells was significantly higher in patients with EOI than those without.^104^

### 3.4 Relationship between CXCR4 expression and prognosis of patients with ALL

Expression of CXCR4 and pCXCR4 in 54 adults with newly diagnosed B‐ALL, including 19 patients with Philadelphia chromosome (Ph), was analysed. CXCR4 expression levels were not related to clinical or laboratory findings or survival. However, pCXCR4 levels are associated with high leukocyte counts, serum bilirubin levels and patients’ outcomes.^105^ The prognostic significance of CXCR4 and VLA‐4 expression was evaluated in 29 adult and 25 paediatric patients with ALL,^106^ and only in adult patients, high CXCR4 expression was associated with shorter disease‐free survival (DFS) and OS and low VLA‐4 expression associated with shorter DFS.^106^

### 3.5 Targeting CXCR4 in treatment of ALL

#### 3.5.1 *Small* *antagonists that targeting CXCR4*

In precursor B ALL and stromal cell co‐cultures, AMD3100 enhanced the cytotoxicity of vincristine and dexamethasone.^89^ Treating murine ALL cells with Ph with low doses of dasatinib over an extended period allowed the emergence of drug‐resistant cells with upregulated CXCR4 expression on their surfaces. A combination of dasatinib and a CXCR4 antagonist resulted in increased cell death,^107^ indicating that this may be a promising strategy to kill ALL cells with Ph. However, it should be noted that CXCR4 antagonists may attenuate the cytotoxicity of cytarabine against ALL cells with MLL rearrangements.^94^ Using human B‐ALL patient‐derived xenograft (PDX) and murine leukaemia models, CXCR4 antagonists have reportedly mobilized ALL cells into PB. Compared with control mice, extended administration of a CXCR4 antagonist to leukaemic mice resulted in a reduction in leukaemia levels in PB and spleens and in the dissemination of ALL cells to extramedullary sites. This is the first study to present the concept of using CXCR4 antagonists to potentiate the effects of chemotherapy.^108^ The mobilization responses of human ALL cells in the PDX model and haematopoietic stem/progenitor cells (HSPCs) in BALB/c mice were compared. ALL cells remained in the circulation for up to 6 hours after AMD3100 administration, when normal HPCs could not be detected. AMD3100 also increased the proportion of actively cycling ALL cells in PB.^109^ The combination of CXCR4 antagonists with tyrosine kinase inhibitor, chemotherapeutic agents and FLT3 inhibitors can effectively eradicate ALL cells in multiple PDX models.^109^, ^110^, ^111^, ^112^ Chemotherapy‐induced upregulation of CXCR4 expression led to drug resistance, which was reversed by AMD3100.^112^ The POE 10‐03 trial enrolled five patients with ALL, but no response was observed.^65^ Consequently, whether patients with ALL can benefit from chemotherapy plus CXCR4 antagonists is unknown, and further studies are needed.

#### 3.5.2 *Other* *treatment strategies based on CXCR4*

CXCR4 may be a target for virotherapy in patients with T‐ALL. A minimized derivative of HIV‐1 was constructed to selectively remove leukaemia cells.^113^ Thereafter, this group constructed doxycycline‐dependent mini‐HIV‐1 variants that may improve the safety of virotherapy.^114^ Ibrutinib may be a potential drug to treat B‐ALL because it can inhibit the phosphorylation of CXCR4 induced by SDF‐1 and the expression and activation of ERK and BCL‐xL.^115^ CXCR4‐targeted endoradiotherapy efficiently reduced leukaemia cells in the T‐ALL PDX model.^116^

**FIGURE 1:**
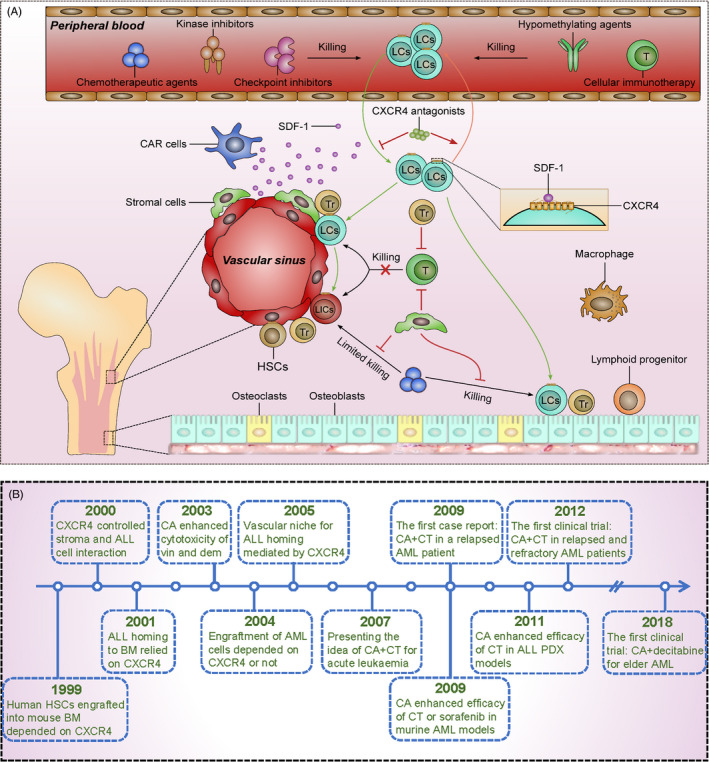
The major progress of research on SDF‐1/CXCR4 in AL. A, SDF‐1/CXCR4 plays important roles in homing and retention of leukaemia cells (LCs) in the BM. SDF‐1 produced by CXCL12‐abundant reticular (CAR) cells and stromal cells can recruit LCs into BM where they reside in special niches. Some LCs reside as leukaemia‐initiating cells (LICs). In the BM microenvironment, Tregs (Tr) and stromal cells protected LCs from killing by effector T cells (T) or chemotherapeutic agents. CXCR4 blockade with special antagonists can mobilize LCs from the protective microenvironment into peripheral blood and render them eradicated by chemotherapeutic agents, kinase inhibitors, checkpoint inhibitors, hypomethylating agents and cellular immunotherapy. B, Shown is the major progress of SDF‐1/CXCR4 function and the potential of CXCR4 as a therapeutic target in AL during the last three decades. CA: CXCR4 antagonists; vin: vincristine; dem: dexamethasone; CT: chemotherapy

In summary, CXCR4 plays very important roles in leukemogenesis and the biological characteristics of AL. CXCR4 is also a prognostic marker and can be used as a target for the treatment of AL. The major progress of research on SDF‐1/CXCR4 in AL is presented in Figure 1.

## 4 ROLE OF CXCR4 IN HSCT

### 4.1 Mobilization of autologous HSCs by AMD3100‐contained regimens

In the preliminary phase I study for evaluating the pharmacokinetics and safety of AMD3100, all participants experienced a dose‐related elevation of the leukocyte counts in PB, indicating that CXCR4 antagonists may have the ability to mobilize HSCs.^117^ For the first time, Liles *et al* studied the mobilization effects of AMD3100 on HSPCs. A single dose of AMD3100 (80 microsubcutaneously) induced rapid and generalized leukocytosis associated with an increase in PB CD34^+^ cells in 10 subjects, identified as pluripotent haematopoietic progenitors by in vitro colony‐forming unit assays.^118^ Subsequently, this group conducted a phase I clinical trial to compare the mobilization responses of G‐CSF and AMD3100 with 18 volunteers. The results showed that AMD3100 significantly increased both G‐CSF‐stimulated mobilization of CD34^+^ cells and leukapheresis yield of CD34^+^ cells, and more T and B cells were observed in AMD3100‐mobilized than G‐CSF‐stimulated leukapheresis products.^119^ Simultaneously, another group demonstrated rapid mobilization of murine and human HSCs and HPCs by AMD3100 and synergistically augmented G‐CSF‐induced mobilization of HPCs, which were proven to be long‐term repopulating cells in subsequent animal experiments.^120^ Based on these observations, Flomenberg *et al* first used G‐CSF plus AMD3100 (G‐CSF +AMD3100) in auto‐HSCT. More CD34^+^ cells were mobilized and target levels of at least 5 × 10^6^ cells/kg for transplantation were completed with fewer apheresis procedures in patients treated with G‐CSF +AMD3100 than those treated with G‐CSF alone. The harvested products also showed long‐term and stable engraftment in subsequent transplantation.^121^ These results were also supported by other studies,^122^, ^123^, ^124^ including a phase III prospective randomized double‐blind placebo‐controlled trial.^125^ Additionally, an obvious increase in primitive PB progenitor cells (PBPCs) with high repopulation capacity was observed in subjects mobilized with G‐CSF +AMD3100 than in those treated with G‐CSF alone.^126^ Compared with PBPCs mobilized by G‐CSF alone, 81 genes were upregulated and 29 genes were downregulated in those treated with G‐CSF +AMD3100. Increased expression was observed in the categories of apoptosis, cell cycle, replication/DNA repair, cell motility and oxygen transport, while decreased expression was found in the proapoptosis gene group and CXCR4 receptor gene itself.^127^ Therefore, the addition of AMD3100 to G‐CSF facilitates the collection of sufficient HSCs for transplantation. AMD3100 was shown to be utilized for mobilizing autologous HSCs in patients with multiple myeloma and lymphoma in 2008 by the FDA. Furthermore, G‐CSF +AMD3100 is an alternative mobilization regimen for patients who fail to collect sufficient HSCs for auto‐HSCT previously mobilized with G‐CSF or G‐CSF plus chemotherapy.^128^, ^129^ This will benefit more patients who have not acquired auto‐HSCT when mobilized with conventional regimens.

### 4.2 Healthy donor mobilization by AMD3100‐contained regimens

Mobilization responses of G‐CSF alone and G‐CSF +AMD3100 for allo‐HSCT in an animal model were compared.^130^ A significantly higher proportion of c‐kit^+^Sca‐1^+^ HSCs and plasmacytoid dendritic cells was found in grafts collected after combinational mobilization than in those mobilized by G‐CSF alone. Recipient mice receiving allografts from G‐CSF +AMD3100 mobilization showed higher mortality associated with increased acute GVHD clinical scores and higher pathology scores in the intestine than those that received G‐CSF grafts, which may be related to the upregulation of CCR6 expression on both CD4^+^ and CD8^+^ T cells.^130^ In 2011, physicians from Italy presented a case report in which G‐CSF +AMD3100 was used for a normal donor of allo‐HSCT who failed marrow harvest due to failure of intubation after anaesthesia. A single leukapheresis yielded sufficient HSCs for transplantation. During the 8 months’ follow‐up, immunosuppressants were withdrawn, and no significant appearance of GVHD was observed.^131^ Patients achieved continuous CR and complete donor cell chimerism.^131^

Mobilization with AMD3100 alone has also been explored. The durable engraftment of AMD3100‐mobilized allogeneic PB mononuclear cells in a canine transplantation model was reported.^132^ In rhesus macaques, AMD3100 could mobilize true long‐term repopulating HSCs with more cells in the G1 phase of the cell cycle and those expressing CXCR4 and VLA‐4 compared with CD34^+^ cells mobilized by G‐CSF.^133^ A comparison of cells mobilized by AMD3100 or G‐CSF was performed in healthy volunteers. AMD3100‐mobilized human mononuclear cells (MNCs) showed enhanced repopulating frequency compared with G‐CSF‐mobilized MNCs from paired donors, and purified CD34^+^ progenitors were at least as efficient as G‐CSF‐mobilized cells in NOD/SCID mice.^134^ The immune characteristics of leukapheresis products mobilized by AMD3100 have been analysed.^135^, ^136^ In the rhesus macaque model, AMD3100 mobilization significantly increased both effector and Treg populations in PB and the resulting leukapheresis products compared with G‐CSF. CD8^+^ T cells (including effector memory T cells) were mobilized to a greater extent than CD4^+^ cells compared with G‐CSF alone.^135^ Considering the high number of effector memory and Tregs in leukapheresis harvests, AMD3100 mobilization may induce less GVHD after allo‐HSCT. T cells mobilized by AMD3100 had a similar phenotype, mixed lymphocyte reactivity, and Foxp3 gene expression levels in CD4^+^ T cells, and expression levels of 84 genes associated with Th1/Th2/Th3 pathways were not altered compared with non‐mobilized T cells. However, G‐CSF mobilization decreased CD62L expression on both CD4^+^ and CD8^+^ T cells and altered the expression of 16 cytokine‐associated genes in CD3^+^ T cells.^136^ In a murine GVHD model, recipient mice that received allografts mobilized by AMD3100 showed a higher incidence of skin GVHD than those receiving G‐CSF mobilized allografts.^136^

The impact of AMD3100 mobilization on engraftment of donor cells and GVHD was evaluated in two clinical trials.^137^, ^138^ In the first, 25 donors were recruited and treated with 240 μg/kg AMD3100, and leukapheresis was performed 4 h later. A total of 22 of 24 donors undergoing 1 or 2 days of leukapheresis had sufficient CD34^+^ cells for transplantation. Finally, 20 patients with haematologic malignancies received allografts and all engrafted neutrophils and platelets. Grade 2 or higher acute GVHD occurred in 35% of patients, and one died of complications related to acute GVHD. All 14 survivors in remission had robust multilineage haematopoiesis and were transfusion‐free.^137^ Another phase II study was conducted using allografts mobilized by AMD3100 alone for sibling donor HSCT. Enough CD34^+^ cells were collected from 63 of 64 donors for transplantation after 1 or 2 days of leukapheresis. The recipients were treated with reduced intensity (RIC) or myeloablative conditioning (MAC). The median time for neutrophil and platelet engraftment was 15 and 18 days, respectively, in patients who received RIC and 13 and 19 days, respectively, in those who received MAC.^138^ Therefore, mobilization with AMD3100 alone seems sufficient for allo‐HSCT.

### 4.3 Other mobilization agents targeting CXCR4

4F‐benzoyl‐TN14003 (BKT140 or T‐140) is a small peptide antagonist of CXCR4 with a stronger affinity than AMD3100 (approximately 21‐fold). BKT140 can mobilize HSPCs, monocytes and B and T lymphocytes into circulation and synergizes with G‐CSF. Compared with AMD3100, BKT140 with or without G‐CSF was significantly more potent in mobilizing HSPCs into PB.^139^ A single injection of BKT140 into healthy volunteers triggered rapid and substantial mobilization of leukocytes and CD34^+^ cells into circulation with intact long‐term engraftment potential, as demonstrated by engraftment of these human cells in NSG immunodeficient mice.^140^ BKT140 administration to mice transplanted with BM cells promoted the production of various progenitors and mature cells and increased the egress of mature cells to the periphery.^141^ BKT140 was combined with chemotherapy and G‐CSF for autologous stem cell mobilization. A single leukapheresis after BKT140 administrated at the highest dose of 0.9 mg/kg achieved 20.6 ± 6.9 × 10^6^/kg CD34^+^ cells for transplantation. The median times of neutrophil and platelet recovery were 12 and 14 days, respectively.^142^ BTK140 with G‐CSF mobilizes autologous HSCs for patients with multiple myeloma in an ongoing phase III, randomized, double‐blind placebo‐control study (NCT03246529).^143^

POL5551, a novel CXCR4 antagonist, showed rapid mobilization kinetics and unprecedented efficiency, exceeding AMD3100 and at higher doses of G‐CSF in mice.^144^ Balixafortide (POL6326), very similar to POL5551, mobilized HSPCs into PB, and mobilization was similar in the dose range 1500‐2500 μg/kg.^145^ ATI‐2341, a potential drug for HSC mobilization, was identified by screening a small CXCR4‐targeted pepducin library.^146^

### 4.4 Blocking CXCR4 in conditioning of allo‐HSCT

In phase I study (n = 12), AMD3100 was used (240 μg/kg) on days −4, −4 to −3, or −4 to −2 in conditioning for second allo‐HSCT in paediatric patients with refractory or relapsed leukaemia. No dose‐limiting toxicity was found, and grade 1 or 2 gastrointestinal side effects were the most common adverse events.^147^ In phase I/II study (n = 45), both G‐CSF (10 μg/kg, days −9 to −4) and AMD3100 (0‐240 μg/kg, days −7 to −4) were added to the conditioning regimen for patients with haematologic malignancies undergoing allo‐HSCT. Compared with historical controls, patients in this study showed increased myeloid chimerism and lower GVHD rates, but no difference in long‐term outcomes.^148^ In another phase I study, 12 patients were enrolled in four sequential cohorts. Patients in the first cohort received one dose of AMD3100 (240 μg/kg) before the first dose of chemotherapeutic agents, and subsequent cohorts received injections before 2, 3, and 4 days of conditioning chemotherapy. All patients were successfully engrafted. Six patients died due to infection (n = 3), relapse (n = 2), or chronic GVHD (n = 1), and the remaining patients maintained continuous CR.^149^ Thus, the addition of CXCR4 antagonists in conditioning was well‐tolerated and associated with increased myeloid chimerism. However, enhanced eradication of residual leukaemia cells or survival benefits were not observed.

### 4.5 Blocking CXCR4 after allo‐HSCT

Because CXCR4 is widely expressed on multiple immune cells, its antagonists will cause tissue redistribution of these cells, which may be relevant to GVHD or GVL effects. AMD3100 can redistribute leukocytes from primary immune organs to peripheral tissues or blood.^150^ The impact of AMD100 on haematopoietic and immune cell reconstitution was evaluated in phase I/II clinical trial (n = 30). AMD3100 was administrated every other day from day +2 to day +21 or until neutrophil recovery. Compared with historic controls, AMD3100 treatment promoted engraftment of neutrophils and platelets, but no significant difference was observed in GVHD occurrence, long‐term outcomes, secretion of inflammatory factors, or immune cell reconstitution.^151^ Using immune‐compromised mice grafted with human B‐ALL generated from human CD34^+^ cells with forced MLL‐AF9 overexpression, we found that injection of AMD3100 after allogeneic lymphocyte infusion could enhance GVL effects, leading to more efficient eradication of leukaemia cells within the immune‐privileged site BM.^152^ Thus, CXCR4 antagonists combined with donor lymphocyte infusion may be a potential treatment option for relapsed patients post‐allo‐HSCT.

**FIGURE 2:**
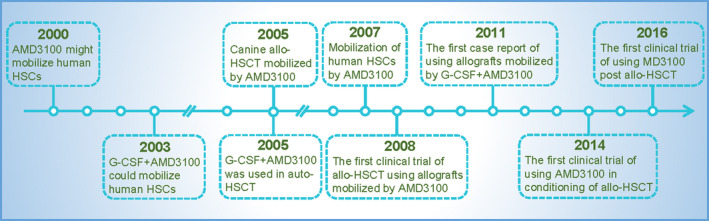
The major progress of research on SDF‐1/CXCR4 in HSCT. Shown is CXCR4 blockade with AMD3100 in mobilization of HSCs, conditioning of allo‐HSCT, and recipients undergoing allo‐HSCT during the last three decades

In summary, CXCR4 antagonists can be used alone or in combination with G‐CSF to mobilize HSCs. Administration of CXCR4 antagonists in conditioning or post‐allo‐HSCT to enhance leukaemia cell eradication by high‐dose chemotherapy or GVL effects was attempted. Further studies or optimized designs may be necessary to improve outcomes. The major progress in research on SDF‐1/CXCR4 in HSCT is shown in Figure 2.

## 5 SUMMARY AND FUTURE DIRECTIONS

The role of CXCR4 in AL has been increasingly complicated with recent research progress. Although the SDF‐1/CXCR4 pathway is critical for the homing to and retention of leukaemia cells in the BM, drug‐resistant MLL‐AF9 AML cells in BM were not affected by AMD3100, and BCP‐ALL cell chemotaxis towards the leukaemia niche was independent of SDF‐1/CXCR4. These results indicate that other mechanisms regulate the migration of leukaemia cells. Targeting CXCR4 by antagonists is a potential therapeutic strategy for AL when combined with chemotherapeutic drugs, kinase inhibitors, hypomethylating agents or checkpoint inhibitors. Blocking CXCR4 with AMD3100 alone or in combination with G‐CSF can be used to mobilize HSCs. New antagonists of CXCR4 are currently being investigated in preclinical studies or clinical trials, and some of them have promising clinical applications.

Combinational therapies consisting of CXCR4 antagonists and chemotherapy have been evaluated in patients, but there have been no randomized clinical trials. Whether patients with high CXCR4 expression are more appropriate for such combinational therapies is unknown. *FLT3*‐ITD mutation is related to high levels of CXCR4 expression, and if a combination of FLT3 inhibitor and CXCR4 antagonists will improve outcomes for these patients needs to be determined. CXCR4 antagonists may mobilize drug‐resistant leukaemia cells or leukaemia‐initiating cells, chemotherapy or molecular target drugs may not kill them effectively, and new combinational strategies still need to be explored. Immunotherapies may be more suitable in combination with CXCR4 antagonists because they are not affected by drug‐resistant leukaemia cells. Post‐transplantation administration of AMD3100 had no significant impact on GVL effects in clinical trials, which may be associated with the time and frequency of drug usage. AMD3100 was administered when immunosuppressants were used, and the intensity was too low. The influence of using CXCR4 antagonists in conditioning regimens or post‐transplantation on outcomes of patients undergoing allo‐HSCT needs to be further verified. Furthermore, donor lymphocyte infusion followed by CXCR4 antagonists may be a promising therapeutic combination for treating relapsed patients after allo‐HSCT or for pre‐emptive treatment in patients with high‐risk diseases or positive minimal residual disease.

## CONFLICT OF INTERESTS

The authors declare no conflict of interest.

## AUTHOR CONTRIBUTION

SL wrote the manuscript. SL, HZ and Y.Y‐G. collected, analysed and summarized the data. SL, HZ and Y.Y‐G. conceptualized this review. Y.Y‐G. and HZ revised the review. The final manuscript was read and approved by all authors.

## Data Availability

The data that support the findings of this study are available from the corresponding author upon reasonable request.

## References

[1] Griffith JW , Sokol CL , Luster AD . Chemokines and chemokine receptors: positioning cells for host defense and immunity. Annu Rev Immunol. 2014;32:659‐702. 24655300 10.1146/annurev-immunol-032713-120145

[2] Nagarsheth N , Wicha MS , Zou W . Chemokines in the cancer microenvironment and their relevance in cancer immunotherapy. Nat Rev Immunol. 2017;17(9):559‐572. 28555670 10.1038/nri.2017.49 PMC5731833

[3] Bleul CC , Fuhlbrigge RC , Casasnovas JM , Aiuti A , Springer TA . A highly efficacious lymphocyte chemoattractant, stromal cell‐derived factor 1 (SDF‐1). J Exp Med. 1996;184(3):1101‐1109. 9064327 10.1084/jem.184.3.1101 PMC2192798

[4] Feng Y , Broder CC , Kennedy PE , Berger EA . HIV‐1 entry cofactor: functional cDNA cloning of a seven‐transmembrane, G protein‐coupled receptor. Science (New York, NY). 1996;272(5263):872‐877. 10.1126/science.272.5263.872 8629022

[5] Janssens R , Struyf S , Proost P . Pathological roles of the homeostatic chemokine CXCL12. Cytokine Growth Factor Rev. 2018;44:51‐68. 30396776 10.1016/j.cytogfr.2018.10.004

[6] Teicher BA , Fricker SP . CXCL12 (SDF‐1)/CXCR4 pathway in cancer. Clin Cancer Res. 2010;16(11):2927‐2931. 20484021 10.1158/1078-0432.CCR-09-2329

[7] Scala S . Molecular pathways: targeting the CXCR4‐CXCL12 axis‐untapped potential in the tumor microenvironment. Clin Cancer Res. 2015;21(19):4278‐4285. 26199389 10.1158/1078-0432.CCR-14-0914

[8] Nagasawa T , Hirota S , Tachibana K , et al. Defects of B‐cell lymphopoiesis and bone‐marrow myelopoiesis in mice lacking the CXC chemokine PBSF/SDF‐1. Nature. 1996;382(6592):635‐638. 8757135 10.1038/382635a0

[9] Zou YR , Kottmann AH , Kuroda M , Taniuchi I , Littman DR . Function of the chemokine receptor CXCR4 in haematopoiesis and in cerebellar development. Nature. 1998;393(6685):595‐599. 9634238 10.1038/31269

[10] Tachibana K , Hirota S , Iizasa H , et al. The chemokine receptor CXCR4 is essential for vascularization of the gastrointestinal tract. Nature. 1998;393(6685):591‐594. 9634237 10.1038/31261

[11] Cao H , Oteiza A , Nilsson SK . Understanding the role of the microenvironment during definitive hemopoietic development. Exp Hematol. 2013;41(9):761‐768. 23800491 10.1016/j.exphem.2013.06.005

[12] Nguyen TS , Lapidot T , Ruf W . Extravascular coagulation in hematopoietic stem and progenitor cell regulation. Blood. 2018;132(2):123‐131. 29866813 10.1182/blood-2017-12-768986 PMC6634957

[13] Rafii S , Cao Z , Lis R , et al. Platelet‐derived SDF‐1 primes the pulmonary capillary vascular niche to drive lung alveolar regeneration. Nat Cell Biol. 2015;17(2):123‐136. 25621952 10.1038/ncb3096 PMC4886751

[14] Wang K , Zhao X , Kuang C , et al. Overexpression of SDF‐1α enhanced migration and engraftment of cardiac stem cells and reduced infarcted size via CXCR4/PI3K pathway. PLoS One. 2012;7(9):e43922. 22984452 10.1371/journal.pone.0043922 PMC3439464

[15] Robin AM , Zhang ZG , Wang L , et al. Stromal cell‐derived factor 1alpha mediates neural progenitor cell motility after focal cerebral ischemia. J Cereb Blood Flow Metab. 2006;26(1):125‐134. 15959456 10.1038/sj.jcbfm.9600172

[16] Kwon SG , Park I , Kwon YW , Lee TW , Park GT , Kim JH . Role of stem cell mobilization in the treatment of ischemic diseases. Arch Pharm Res. 2019;42(3):224‐231. 30680545 10.1007/s12272-019-01123-2

[17] Oberlin E , Amara A , Bachelerie F , et al. The CXC chemokine SDF‐1 is the ligand for LESTR/fusin and prevents infection by T‐cell‐line‐adapted HIV‐1. Nature. 1996;382(6594):833‐835. 8752281 10.1038/382833a0

[18] Zhang C , Zhu R , Cao Q , Yang X , Huang Z , An J . Discoveries and developments of CXCR4‐targeted HIV‐1 entry inhibitors. Exp Biol Med (Maywood, NJ). 2020;245(5):477‐485. 10.1177/1535370220901498 PMC7082890 32019336

[19] Chatterjee S , Behnam Azad B , Nimmagadda S . The intricate role of CXCR4 in cancer. Adv Cancer Res. 2014;124:31‐82. 25287686 10.1016/B978-0-12-411638-2.00002-1 PMC4322894

[20] Domanska UM , Kruizinga RC , Nagengast WB , et al. A review on CXCR4/CXCL12 axis in oncology: no place to hide. Eur J Cancer. 2013;49(1):219‐230. 22683307 10.1016/j.ejca.2012.05.005

[21] Maroni P , Bendinelli P , Matteucci E , Desiderio MA . HGF induces CXCR4 and CXCL12‐mediated tumor invasion through Ets1 and NF‐kappaB. Carcinogenesis. 2007;28(2):267‐279. 16840440 10.1093/carcin/bgl129

[22] Krohn A , Song YH , Muehlberg F , Droll L , Beckmann C , Alt E . CXCR4 receptor positive spheroid forming cells are responsible for tumor invasion in vitro. Cancer Lett. 2009;280(1):65‐71. 19286309 10.1016/j.canlet.2009.02.005

[23] Du R , Lu KV , Petritsch C , et al. HIF1alpha induces the recruitment of bone marrow‐derived vascular modulatory cells to regulate tumor angiogenesis and invasion. Cancer Cell. 2008;13(3):206‐220. 18328425 10.1016/j.ccr.2008.01.034 PMC2643426

[24] Derlin T , Hueper K . CXCR4‐targeted therapy in breast cancer. Lancet Oncol. 2018;19(8):e370. 30102220 10.1016/S1470-2045(18)30480-7

[25] Chen IX , Chauhan VP , Posada J , et al. Blocking CXCR4 alleviates desmoplasia, increases T‐lymphocyte infiltration, and improves immunotherapy in metastatic breast cancer. Proc Natl Acad Sci USA. 2019;116(10):4558‐4566. 30700545 10.1073/pnas.1815515116 PMC6410779

[26] Seo YD , Jiang X , Sullivan KM , et al. Mobilization of CD8(+) T cells via CXCR4 blockade facilitates PD‐1 checkpoint therapy in human pancreatic cancer. Clin Cancer Res. 2019;25(13):3934‐3945. 30940657 10.1158/1078-0432.CCR-19-0081 PMC6606359

[27] Hong Z , Wei Z , Xie T , et al. Targeting chemokines for acute lymphoblastic leukemia therapy. J Hematol Oncol. 2021;14(1):48. 33743810 10.1186/s13045-021-01060-y PMC7981899

[28] Shallis RM , Wang R , Davidoff A , Ma X , Zeidan AM . Epidemiology of acute myeloid leukemia: recent progress and enduring challenges. Blood Rev. 2019;36:70‐87. 31101526 10.1016/j.blre.2019.04.005

[29] Yanada M , Takeuchi J , Sugiura I , et al. High complete remission rate and promising outcome by combination of imatinib and chemotherapy for newly diagnosed BCR‐ABL‐positive acute lymphoblastic leukemia: a phase II study by the Japan Adult Leukemia Study Group. J Clin Oncol. 2006;24(3):460‐466. 16344315 10.1200/JCO.2005.03.2177

[30] Maude SL , Frey N , Shaw PA , et al. Chimeric antigen receptor T cells for sustained remissions in leukemia. N Engl J Med. 2014;371(16):1507‐1517. 25317870 10.1056/NEJMoa1407222 PMC4267531

[31] Stone RM , Mandrekar SJ , Sanford BL , et al. Midostaurin plus chemotherapy for acute myeloid leukemia with a FLT3 mutation. N Engl J Med. 2017;377(5):454‐464. 28644114 10.1056/NEJMoa1614359 PMC5754190

[32] Peled A , Petit I , Kollet O , et al. Dependence of human stem cell engraftment and repopulation of NOD/SCID mice on CXCR4. Science (New York, NY). 1999;283(5403):845‐848. 10.1126/science.283.5403.845 9933168

[33] Voermans C , Kooi ML , Rodenhuis S , van der Lelie H , van der Schoot CE , Gerritsen WR . In vitro migratory capacity of CD34+ cells is related to hematopoietic recovery after autologous stem cell transplantation. Blood. 2001;97(3):799‐804. 11157500 10.1182/blood.v97.3.799

[34] Voermans C , van Heese WP , de Jong I , Gerritsen WR , van Der Schoot CE . Migratory behavior of leukemic cells from acute myeloid leukemia patients. Leukemia. 2002;16(4):650‐657. 11960346 10.1038/sj.leu.2402431

[35] Monaco G , Konopleva M , Munsell M , et al. Engraftment of acute myeloid leukemia in NOD/SCID mice is independent of CXCR4 and predicts poor patient survival. Stem Cells (Dayton, Ohio). 2004;22(2):188‐201. 10.1634/stemcells.22-2-188 14990858

[36] Tavor S , Petit I , Porozov S , et al. CXCR4 regulates migration and development of human acute myelogenous leukemia stem cells in transplanted NOD/SCID mice. Can Res. 2004;64(8):2817‐2824. 10.1158/0008-5472.can-03-3693 15087398

[37] Monaco G , Belmont JW , Konopleva M , et al. Correlation between CXCR4 and homing or engraftment of acute myelogenous leukemia. Can Res. 2004;64(18):6832. Author reply 6832‐6833. 10.1158/0008-5472.CAN-04-1936 15375005

[38] Ramakrishnan R , Peña‐Martínez P , Agarwal P , et al. CXCR4 Signaling has a CXCL12‐independent essential role in murine MLL‐AF9‐driven acute myeloid leukemia. Cell Rep. 2020;31(8):107684. 32460032 10.1016/j.celrep.2020.107684 PMC8109054

[39] Duarte D , Amarteifio S , Ang H , et al. Defining the in vivo characteristics of acute myeloid leukemia cells behavior by intravital imaging. Immunol Cell Biol. 2019;97(2):229‐235. 30422351 10.1111/imcb.12216 PMC6446728

[40] Fiegl M , Samudio I , Clise‐Dwyer K , Burks JK , Mnjoyan Z , Andreeff M . CXCR4 expression and biologic activity in acute myeloid leukemia are dependent on oxygen partial pressure. Blood. 2009;113(7):1504‐1512. 18957686 10.1182/blood-2008-06-161539 PMC2644078

[41] Shao HY , Miao ZY , Hui C , et al. Nucleophosmin gene mutations promote NIH3T3 cell migration and invasion through CXCR4 and MMPs. Exp Mol Pathol. 2011;90(1):38‐44. 21122805 10.1016/j.yexmp.2010.11.009

[42] Mannelli F , Cutini I , Gianfaldoni G , et al. CXCR4 expression accounts for clinical phenotype and outcome in acute myeloid leukemia. Cytometry B, Clin Cytom. 2014;86(5):340‐349. 24500843 10.1002/cyto.b.21156

[43] Konoplev S , Lin P , Yin CC , et al. CXC chemokine receptor 4 expression, CXC chemokine receptor 4 activation, and wild‐type nucleophosmin are independently associated with unfavorable prognosis in patients with acute myeloid leukemia. Clin Lymphoma Myeloma Leuk. 2013;13(6):686‐692. 24035716 10.1016/j.clml.2013.05.013 PMC4206258

[44] Kuo YY , Hou HA , Chen YK , et al. The N‐terminal CEBPA mutant in acute myeloid leukemia impairs CXCR4 expression. Haematologica. 2014;99(12):1799‐1807. 25193961 10.3324/haematol.2014.107821 PMC4258761

[45] Cao T , Jiang N , Liao H , Shuai X , Su J , Zheng Q . The FLT3‐ITD mutation and the expression of its downstream signaling intermediates STAT5 and Pim‐1 are positively correlated with CXCR4 expression in patients with acute myeloid leukemia. Sci Rep. 2019;9(1):12209. 31434952 10.1038/s41598-019-48687-z PMC6704161

[46] Rombouts EJ , Pavic B , Löwenberg B , Ploemacher RE . Relation between CXCR‐4 expression, Flt3 mutations, and unfavorable prognosis of adult acute myeloid leukemia. Blood. 2004;104(2):550‐557. 15054042 10.1182/blood-2004-02-0566

[47] Du W , Lu C , Zhu X , et al. Prognostic significance of CXCR4 expression in acute myeloid leukemia. Cancer Med. 2019;8(15):6595‐6603. 31518054 10.1002/cam4.2535 PMC6825984

[48] Spinello I , Quaranta MT , Riccioni R , et al. MicroRNA‐146a and AMD3100, two ways to control CXCR4 expression in acute myeloid leukemias. Blood Cancer J. 2011;1(6):e26. 22829170 10.1038/bcj.2011.24 PMC3255264

[49] Sison EA , McIntyre E , Magoon D , Brown P . Dynamic chemotherapy‐induced upregulation of CXCR4 expression: a mechanism of therapeutic resistance in pediatric AML. Mol Cancer Res. 2013;11(9):1004‐1016. 23754844 10.1158/1541-7786.MCR-13-0114 PMC3778118

[50] Spoo AC , Lübbert M , Wierda WG , Burger JA . CXCR4 is a prognostic marker in acute myelogenous leukemia. Blood. 2007;109(2):786‐791. 16888090 10.1182/blood-2006-05-024844

[51] Konoplev S , Rassidakis GZ , Estey E , et al. Overexpression of CXCR4 predicts adverse overall and event‐free survival in patients with unmutated FLT3 acute myeloid leukemia with normal karyotype. Cancer. 2007;109(6):1152‐1156. 17315232 10.1002/cncr.22510

[52] Ahn JY , Seo K , Weinberg OK , Arber DA . The prognostic value of CXCR4 in acute myeloid leukemia. Appl Immunohistochem Mol Morphol. 2013;21(1):79‐84. 22914607 10.1097/PAI.0b013e3182606f4d

[53] Bae MH , Oh SH , Park CJ , et al. VLA‐4 and CXCR4 expression levels show contrasting prognostic impact (favorable and unfavorable, respectively) in acute myeloid leukemia. Ann Hematol. 2015;94(10):1631‐1638. 26155911 10.1007/s00277-015-2442-8

[54] Matsuo H , Nakamura N , Tomizawa D , et al. CXCR4 overexpression is a poor prognostic factor in pediatric acute myeloid leukemia with low risk: a report from the Japanese Pediatric Leukemia/Lymphoma Study Group. Pediatr Blood Cancer. 2016;63(8):1394‐1399. 27135782 10.1002/pbc.26035

[55] Liesveld JL , Bechelli J , Rosell K , et al. Effects of AMD3100 on transmigration and survival of acute myelogenous leukemia cells. Leuk Res. 2007;31(11):1553‐1563. 17403536 10.1016/j.leukres.2007.02.017 PMC2133372

[56] Tavor S , Eisenbach M , Jacob‐Hirsch J , et al. The CXCR4 antagonist AMD3100 impairs survival of human AML cells and induces their differentiation. Leukemia. 2008;22(12):2151‐5158. 18769446 10.1038/leu.2008.238

[57] Zeng Z , Shi YX , Samudio IJ , et al. Targeting the leukemia microenvironment by CXCR4 inhibition overcomes resistance to kinase inhibitors and chemotherapy in AML. Blood. 2009;113(24):6215‐6224. 18955566 10.1182/blood-2008-05-158311 PMC2699240

[58] Nervi B , Ramirez P , Rettig MP , et al. Chemosensitization of acute myeloid leukemia (AML) following mobilization by the CXCR4 antagonist AMD3100. Blood. 2009;113(24):6206‐6214. 19050309 10.1182/blood-2008-06-162123 PMC2699239

[59] Jacobi A , Thieme S , Lehmann R , et al. Impact of CXCR4 inhibition on FLT3‐ITD‐positive human AML blasts. Exp Hematol. 2010;38(3):180‐190. 20035824 10.1016/j.exphem.2009.12.003 PMC4777334

[60] Zhang Y , Patel S , Abdelouahab H , et al. CXCR4 inhibitors selectively eliminate CXCR4‐expressing human acute myeloid leukemia cells in NOG mouse model. Cell Death Dis. 2012;3(10):e396. 23034331 10.1038/cddis.2012.137 PMC3481125

[61] Hwang HS , Han AR , Lee JY , Park GS , Min WS , Kim HJ . Enhanced anti‐leukemic effects through induction of immunomodulating microenvironment by blocking CXCR4 and PD‐L1 in an AML mouse model. Immunol Invest. 2019;48(1):96‐105. 30204524 10.1080/08820139.2018.1497057

[62] Fierro FA , Brenner S , Oelschlaegel U , et al. Combining SDF‐1/CXCR4 antagonism and chemotherapy in relapsed acute myeloid leukemia. Leukemia. 2009;23(2):393‐396. 18615106 10.1038/leu.2008.182

[63] Uy GL , Rettig MP , Motabi IH , et al. A phase 1/2 study of chemosensitization with the CXCR4 antagonist plerixafor in relapsed or refractory acute myeloid leukemia. Blood. 2012;119(17):3917‐3924. 22308295 10.1182/blood-2011-10-383406 PMC3350358

[64] Uy GL , Rettig MP , Stone RM , et al. A phase 1/2 study of chemosensitization with plerixafor plus G‐CSF in relapsed or refractory acute myeloid leukemia. Blood Cancer J. 2017;7(3):e542. 28282031 10.1038/bcj.2017.21 PMC5380905

[65] Cooper TM , Sison EAR , Baker SD , et al. A phase 1 study of the CXCR4 antagonist plerixafor in combination with high‐dose cytarabine and etoposide in children with relapsed or refractory acute leukemias or myelodysplastic syndrome: a Pediatric Oncology Experimental Therapeutics Investigators' Consortium study (POE 10–03). Pediatr Blood Cancer. 2017;64(8):e26414. 10.1002/pbc.26414 PMC5675008 28409853

[66] Martínez‐Cuadrón D , Boluda B , Martínez P , et al. A phase I‐II study of plerixafor in combination with fludarabine, idarubicin, cytarabine, and G‐CSF (PLERIFLAG regimen) for the treatment of patients with the first early‐relapsed or refractory acute myeloid leukemia. Ann Hematol. 2018;97(5):763‐772. 29392425 10.1007/s00277-018-3229-5

[67] Roboz GJ , Ritchie EK , Dault Y , et al. Phase I trial of plerixafor combined with decitabine in newly diagnosed older patients with acute myeloid leukemia. Haematologica. 2018;103(8):1308‐1316. 29724902 10.3324/haematol.2017.183418 PMC6068018

[68] Zeng Z , Samudio IJ , Munsell M , et al. Inhibition of CXCR4 with the novel RCP168 peptide overcomes stroma‐mediated chemoresistance in chronic and acute leukemias. Mol Cancer Ther. 2006;5(12):3113‐3121. 17172414 10.1158/1535-7163.MCT-06-0228

[69] Li X , Guo H , Yang Y , et al. A designed peptide targeting CXCR4 displays anti‐acute myelocytic leukemia activity in vitro and in vivo. Sci Rep. 2014;4:6610. 25312253 10.1038/srep06610 PMC4196105

[70] Li X , Guo H , Duan H , et al. Improving chemotherapeutic efficiency in acute myeloid leukemia treatments by chemically synthesized peptide interfering with CXCR4/CXCL12 axis. Sci Rep. 2015;5:16228. 26538086 10.1038/srep16228 PMC4633653

[71] Meng J , Ge Y , Xing H , et al. Synthetic CXCR4 antagonistic peptide assembling with nanoscaled micelles combat acute myeloid leukemia. Small. 2020;16(31):e2001890. 32608185 10.1002/smll.202001890

[72] Cho BS , Zeng Z , Mu H , et al. Antileukemia activity of the novel peptidic CXCR4 antagonist LY2510924 as monotherapy and in combination with chemotherapy. Blood. 2015;126(2):222‐232. 26031918 10.1182/blood-2015-02-628677 PMC4497963

[73] Kim BR , Jung SH , Han AR , et al. CXCR4 inhibition enhances efficacy of FLT3 inhibitors in FLT3‐mutated AML augmented by suppressed TGF‐b signaling. Cancers. 2020;12(7):1737. 10.3390/cancers12071737 PMC7407511 32629802

[74] Boddu P , Borthakur G , Koneru M , et al. Initial report of a phase I study of LY2510924, idarubicin, and cytarabine in relapsed/refractory acute myeloid leukemia. Front Oncol. 2018;8:369. 30319961 10.3389/fonc.2018.00369 PMC6167965

[75] Liu SH , Gu Y , Pascual B , et al. A novel CXCR4 antagonist IgG1 antibody (PF‐06747143) for the treatment of hematologic malignancies. Blood Adv. 2017;1(15):1088‐1100. 29296751 10.1182/bloodadvances.2016003921 PMC5728311

[76] Zhang Y , Saavedra E , Tang R , et al. Targeting primary acute myeloid leukemia with a new CXCR4 antagonist IgG1 antibody (PF‐06747143). Sci Rep. 2017;7(1):7305. 28779088 10.1038/s41598-017-07848-8 PMC5544749

[77] Abraham M , Klein S , Bulvik B , et al. The CXCR4 inhibitor BL‐8040 induces the apoptosis of AML blasts by downregulating ERK, BCL‐2, MCL‐1 and cyclin‐D1 via altered miR‐15a/16‐1 expression. Leukemia. 2017;31(11):2336‐2346. 28280274 10.1038/leu.2017.82

[78] Xu Y , Duggineni S , Espitia S , Richman DD , An J , Huang Z . A synthetic bivalent ligand of CXCR4 inhibits HIV infection. Biochem Biophys Res Comm. 2013;435(4):646‐650. 23688427 10.1016/j.bbrc.2013.05.038 PMC3752463

[79] Huang Y , Huang Z , An J , Xu Y . A novel dimeric CXCR4 antagonist synergizes with chemotherapy in acute myeloid leukaemia by mobilizing leukaemic cells from their associated bone marrow niches. Br J Haematol. 2019;187(1):e11‐e15. 31388999 10.1111/bjh.16127

[80] Zaitseva L , Murray MY , Shafat MS , et al. Ibrutinib inhibits SDF1/CXCR4 mediated migration in AML. Oncotarget. 2014;5(20):9930‐9938. 25294819 10.18632/oncotarget.2479 PMC4259448

[81] Landry B , Gül‐Uludağ H , Plianwong S , et al. Targeting CXCR4/SDF‐1 axis by lipopolymer complexes of siRNA in acute myeloid leukemia. J Controlled Release. 2016;224:8‐21. 10.1016/j.jconrel.2015.12.052 26742943

[82] Wang Y , Xie Y , Williams J , et al. Use of polymeric CXCR4 inhibitors as siRNA delivery vehicles for the treatment of acute myeloid leukemia. Cancer Gene Ther. 2020;27(1–2):45‐55. 31028289 10.1038/s41417-019-0095-9

[83] Pallarès V , Unzueta U , Falgàs A , et al. An Auristatin nanoconjugate targeting CXCR4+ leukemic cells blocks acute myeloid leukemia dissemination. J Hematol Oncol. 2020;13(1):36. 32295630 10.1186/s13045-020-00863-9 PMC7160905

[84] Wan Y , Zhang C , Xu Y , et al. Hyperfunction of CD4 CD25 regulatory T cells in de novo acute myeloid leukemia. BMC Cancer. 2020;20(1):472. 32456622 10.1186/s12885-020-06961-8 PMC7249438

[85] Wang R , Feng W , Wang H , et al. Blocking migration of regulatory T cells to leukemic hematopoietic microenvironment delays disease progression in mouse leukemia model. Cancer Lett. 2020;469:151‐161. 31669202 10.1016/j.canlet.2019.10.032

[86] Zhou J , Hu L , Cui Z , et al. Interaction of SDF‐1alpha and CXCR4 plays an important role in pulmonary cellular infiltration in differentiation syndrome. Int J Hematol. 2010;91(2):293‐302. 20084476 10.1007/s12185-009-0488-x

[87] Bradstock KF , Makrynikola V , Bianchi A , Shen W , Hewson J , Gottlieb DJ . Effects of the chemokine stromal cell‐derived factor‐1 on the migration and localization of precursor‐B acute lymphoblastic leukemia cells within bone marrow stromal layers. Leukemia. 2000;14(5):882‐888. 10803521 10.1038/sj.leu.2401729

[88] Möhle R , Schittenhelm M , Failenschmid C , et al. Functional response of leukaemic blasts to stromal cell‐derived factor‐1 correlates with preferential expression of the chemokine receptor CXCR4 in acute myelomonocytic and lymphoblastic leukaemia. Br J Haematol. 2000;110(3):563‐572. 10997965 10.1046/j.1365-2141.2000.02157.x

[89] Juarez J , Bradstock KF , Gottlieb DJ , Bendall LJ . Effects of inhibitors of the chemokine receptor CXCR4 on acute lymphoblastic leukemia cells in vitro. Leukemia. 2003;17(7):1294‐1300. 12835717 10.1038/sj.leu.2402998

[90] Shen W , Bendall LJ , Gottlieb DJ , Bradstock KF . The chemokine receptor CXCR4 enhances integrin‐mediated in vitro adhesion and facilitates engraftment of leukemic precursor‐B cells in the bone marrow. Exp Hematol. 2001;29(12):1439‐1447. 11750103 10.1016/s0301-472x(01)00741-x

[91] Sipkins DA , Wei X , Wu JW , et al. In vivo imaging of specialized bone marrow endothelial microdomains for tumour engraftment. Nature. 2005;435(7044):969‐973. 15959517 10.1038/nature03703 PMC2570168

[92] Pitt LA , Tikhonova AN , Hu H , et al. CXCL12‐producing vascular endothelial niches control acute T cell leukemia maintenance. Cancer Cell. 2015;27(6):755‐768. 26058075 10.1016/j.ccell.2015.05.002 PMC4461838

[93] Passaro D , Irigoyen M , Catherinet C , et al. CXCR4 Is required for leukemia‐initiating cell activity in T cell acute lymphoblastic leukemia. Cancer Cell. 2015;27(6):769‐779. 26058076 10.1016/j.ccell.2015.05.003

[94] Ando N , Furuichi Y , Kasai S , et al. Chemosensitivity is differentially regulated by the SDF‐1/CXCR4 and SDF‐1/CXCR7 axes in acute lymphoblastic leukemia with MLL gene rearrangements. Leuk Res. 2018;75:36‐44. 30453100 10.1016/j.leukres.2018.11.001

[95] Spiegel A , Kollet O , Peled A , et al. Unique SDF‐1‐induced activation of human precursor‐B ALL cells as a result of altered CXCR4 expression and signaling. Blood. 2004;103(8):2900‐2907. 15070661 10.1182/blood-2003-06-1891

[96] Bendall LJ , Baraz R , Juarez J , Shen W , Bradstock KF . Defective p38 mitogen‐activated protein kinase signaling impairs chemotaxic but not proliferative responses to stromal‐derived factor‐1alpha in acute lymphoblastic leukemia. Can Res. 2005;65(8):3290‐3298. 10.1158/0008-5472.CAN-04-3402 15833862

[97] Juarez JG , Thien M , Dela Pena A , Baraz R , Bradstock KF , Bendall LJ . CXCR4 mediates the homing of B cell progenitor acute lymphoblastic leukaemia cells to the bone marrow via activation of p38MAPK. Br J Haematol. 2009;145(4):491‐499. 19344405 10.1111/j.1365-2141.2009.07648.x

[98] de Rooij B , Polak R , van den Berk LCJ , Stalpers F , Pieters R , den Boer ML . Acute lymphoblastic leukemia cells create a leukemic niche without affecting the CXCR4/CXCL12 axis. Haematologica. 2017;102(10):e389‐e393. 28619846 10.3324/haematol.2016.159517 PMC5622868

[99] Dürig J , Schmücker U , Dührsen U . Differential expression of chemokine receptors in B cell malignancies. Leukemia. 2001;15(5):752‐756. 11368435 10.1038/sj.leu.2402107

[100] van den Berk LC , van der Veer A , Willemse ME , et al. Disturbed CXCR4/CXCL12 axis in paediatric precursor B‐cell acute lymphoblastic leukaemia. Br J Haematol. 2014;166(2):240‐249. 24697337 10.1111/bjh.12883

[101] Wu S , Gessner R , Taube T , et al. Chemokine IL‐8 and chemokine receptor CXCR3 and CXCR4 gene expression in childhood acute lymphoblastic leukemia at first relapse. J Pediatr Hematol Oncol. 2006;28(4):216‐220. 16679918 10.1097/01.mph.0000212908.14642.a5

[102] Freret M , Gouel F , Buquet C , et al. Rac‐1 GTPase controls the capacity of human leukaemic lymphoblasts to migrate on fibronectin in response to SDF‐1α (CXCL12). Leuk Res. 2011;35(7):971‐973. 21458858 10.1016/j.leukres.2011.03.011

[103] Crazzolara R , Jöhrer K , Johnstone RW , et al. Histone deacetylase inhibitors potently repress CXCR4 chemokine receptor expression and function in acute lymphoblastic leukaemia. Br J Haematol. 2002;119(4):965‐969. 12472574 10.1046/j.1365-2141.2002.03955.x

[104] Crazzolara R , Kreczy A , Mann G , et al. High expression of the chemokine receptor CXCR4 predicts extramedullary organ infiltration in childhood acute lymphoblastic leukaemia. Br J Haematol. 2001;115(3):545‐553. 11736934 10.1046/j.1365-2141.2001.03164.x

[105] Konoplev S , Jorgensen JL , Thomas DA , et al. Phosphorylated CXCR4 is associated with poor survival in adults with B‐acute lymphoblastic leukemia. Cancer. 2011;117(20):4689‐4695. 21456010 10.1002/cncr.26113

[106] Ko SY , Park CJ , Park SH , et al. High CXCR4 and low VLA‐4 expression predicts poor survival in adults with acute lymphoblastic leukemia. Leuk Res. 2014;38(1):65‐70. 24239175 10.1016/j.leukres.2013.10.016

[107] Fei F , Stoddart S , Müschen M , Kim YM , Groffen J , Heisterkamp N . Development of resistance to dasatinib in Bcr/Abl‐positive acute lymphoblastic leukemia. Leukemia. 2010;24(4):813‐820. 20111071 10.1038/leu.2009.302 PMC3038787

[108] Juarez J , Dela Pena A , Baraz R , et al. CXCR4 antagonists mobilize childhood acute lymphoblastic leukemia cells into the peripheral blood and inhibit engraftment. Leukemia. 2007;21(6):1249‐1257. 17410186 10.1038/sj.leu.2404684

[109] Welschinger R , Liedtke F , Basnett J , et al. Plerixafor (AMD3100) induces prolonged mobilization of acute lymphoblastic leukemia cells and increases the proportion of cycling cells in the blood in mice. Exp Hematol. 2013;41(3):293‐302.e291. 23178377 10.1016/j.exphem.2012.11.004

[110] Parameswaran R , Yu M , Lim M , Groffen J , Heisterkamp N . Combination of drug therapy in acute lymphoblastic leukemia with a CXCR4 antagonist. Leukemia. 2011;25(8):1314‐1323. 21483439 10.1038/leu.2011.76 PMC3135709

[111] Sison EA , Rau RE , McIntyre E , Li L , Small D , Brown P . MLL‐rearranged acute lymphoblastic leukaemia stem cell interactions with bone marrow stroma promote survival and therapeutic resistance that can be overcome with CXCR4 antagonism. Br J Haematol. 2013;160(6):785‐797. 23294096 10.1111/bjh.12205 PMC4005340

[112] Sison EA , Magoon D , Li L , et al. Plerixafor as a chemosensitizing agent in pediatric acute lymphoblastic leukemia: efficacy and potential mechanisms of resistance to CXCR4 inhibition. Oncotarget. 2014;5(19):8947‐8958. 25333254 10.18632/oncotarget.2407 PMC4253409

[113] Jeeninga RE , Jan B , van der Linden B , van den Berg H , Berkhout B . Construction of a minimal HIV‐1 variant that selectively replicates in leukemic derived T‐cell lines: towards a new virotherapy approach. Can Res. 2005;65(8):3347‐3355. 10.1158/0008-5472.CAN-04-4280 15833868

[114] Jeeninga RE , Jan B , van den Berg H , Berkhout B . Construction of doxycyline‐dependent mini‐HIV‐1 variants for the development of a virotherapy against leukemias. Retrovirology. 2006;3:64. 17005036 10.1186/1742-4690-3-64 PMC1592508

[115] Hu YY , Tao SD , Ma JJ , Zhou LT , Chen Y , Yu L . SDF‐1α/CXCR4 mediated drug resistance can be reversed by ibrutinib in acute lymphoblastic leukemia. Zhongguo shi yan xue ye xue za zhi. 2017;25(3):754‐760. 28641630 10.7534/j.issn.1009-2137.2017.03.021

[116] Habringer S , Lapa C , Herhaus P , et al. Dual targeting of acute leukemia and supporting Niche by CXCR4‐directed theranostics. Theranostics. 2018;8(2):369‐383. 29290814 10.7150/thno.21397 PMC5743554

[117] Hendrix CW , Flexner C , MacFarland RT , et al. Pharmacokinetics and safety of AMD‐3100, a novel antagonist of the CXCR‐4 chemokine receptor, in human volunteers. Antimicrob Agents Chemother. 2000;44(6):1667‐1673. 10817726 10.1128/aac.44.6.1667-1673.2000 PMC89930

[118] Liles WC , Broxmeyer HE , Rodger E , et al. Mobilization of hematopoietic progenitor cells in healthy volunteers by AMD3100, a CXCR4 antagonist. Blood. 2003;102(8):2728‐2730. 12855591 10.1182/blood-2003-02-0663

[119] Liles WC , Rodger E , Broxmeyer HE , et al. Augmented mobilization and collection of CD34+ hematopoietic cells from normal human volunteers stimulated with granulocyte‐colony‐stimulating factor by single‐dose administration of AMD3100, a CXCR4 antagonist. Transfusion. 2005;45(3):295‐300. 15752146 10.1111/j.1537-2995.2005.04222.x

[120] Broxmeyer HE , Orschell CM , Clapp DW , et al. Rapid mobilization of murine and human hematopoietic stem and progenitor cells with AMD3100, a CXCR4 antagonist. J Exp Med. 2005;201(8):1307‐1318. 15837815 10.1084/jem.20041385 PMC2213145

[121] Flomenberg N , Devine SM , Dipersio JF , et al. The use of AMD3100 plus G‐CSF for autologous hematopoietic progenitor cell mobilization is superior to G‐CSF alone. Blood. 2005;106(5):1867‐1874. 15890685 10.1182/blood-2005-02-0468

[122] Gazitt Y , Freytes CO , Akay C , Badel K , Calandra G . Improved mobilization of peripheral blood CD34+ cells and dendritic cells by AMD3100 plus granulocyte‐colony‐stimulating factor in non‐Hodgkin's lymphoma patients. Stem Cells Dev. 2007;16(4):657‐666. 17784839 10.1089/scd.2006.0087

[123] Cashen A , Lopez S , Gao F , et al. A phase II study of plerixafor (AMD3100) plus G‐CSF for autologous hematopoietic progenitor cell mobilization in patients with Hodgkin lymphoma. Biol Blood Marrow Transplant. 2008;14(11):1253‐1261. 18940680 10.1016/j.bbmt.2008.08.011

[124] Dugan MJ , Maziarz RT , Bensinger WI , et al. Safety and preliminary efficacy of plerixafor (Mozobil) in combination with chemotherapy and G‐CSF: an open‐label, multicenter, exploratory trial in patients with multiple myeloma and non‐Hodgkin's lymphoma undergoing stem cell mobilization. Bone Marrow Transplant. 2010;45(1):39‐47. 19483760 10.1038/bmt.2009.119

[125] DiPersio JF , Micallef IN , Stiff PJ , et al. Phase III prospective randomized double‐blind placebo‐controlled trial of plerixafor plus granulocyte colony‐stimulating factor compared with placebo plus granulocyte colony‐stimulating factor for autologous stem‐cell mobilization and transplantation for patients with non‐Hodgkin's lymphoma. J Clin Oncol. 2009;27(28):4767‐4773. 19720922 10.1200/JCO.2008.20.7209

[126] Fruehauf S , Veldwijk MR , Seeger T , et al. A combination of granulocyte‐colony‐stimulating factor (G‐CSF) and plerixafor mobilizes more primitive peripheral blood progenitor cells than G‐CSF alone: results of a European phase II study. Cytotherapy. 2009;11(8):992‐1001. 19929463 10.3109/14653240903121245

[127] Fruehauf S , Seeger T , Maier P , et al. The CXCR4 antagonist AMD3100 releases a subset of G‐CSF‐primed peripheral blood progenitor cells with specific gene expression characteristics. Exp Hematol. 2006;34(8):1052‐1059. 16863911 10.1016/j.exphem.2006.06.003

[128] Fowler CJ , Dunn A , Hayes‐Lattin B , et al. Rescue from failed growth factor and/or chemotherapy HSC mobilization with G‐CSF and plerixafor (AMD3100): an institutional experience. Bone Marrow Transplant. 2009;43(12):909‐917. 19182831 10.1038/bmt.2008.409

[129] Worel N , Rosskopf K , Neumeister P , et al. Plerixafor and granulocyte‐colony‐stimulating factor (G‐CSF) in patients with lymphoma and multiple myeloma previously failing mobilization with G‐CSF with or without chemotherapy for autologous hematopoietic stem cell mobilization: the Austrian experience on a named patient program. Transfusion. 2011;51(5):968‐975. 20880037 10.1111/j.1537-2995.2010.02896.x

[130] Arbez J , Saas P , Lamarthée B , et al. Impact of donor hematopoietic cells mobilized with G‐CSF and plerixafor on murine acute graft‐versus‐host‐disease. Cytotherapy. 2015;17(7):948‐955. 25813681 10.1016/j.jcyt.2015.02.009

[131] Leotta S , Poidomani M , Mauro E , Spadaro A , Marturano E , Milone G . AMD3100 for urgent PBSC mobilization and allogeneic transplantation from a normal donor after failed marrow harvest. Bone Marrow Transplant. 2011;46(2):314‐316. 20436520 10.1038/bmt.2010.98

[132] Burroughs L , Mielcarek M , Little MT , et al. Durable engraftment of AMD3100‐mobilized autologous and allogeneic peripheral‐blood mononuclear cells in a canine transplantation model. Blood. 2005;106(12):4002‐4008. 16105977 10.1182/blood-2005-05-1937 PMC1895108

[133] Larochelle A , Krouse A , Metzger M , et al. AMD3100 mobilizes hematopoietic stem cells with long‐term repopulating capacity in nonhuman primates. Blood. 2006;107(9):3772‐3778. 16439684 10.1182/blood-2005-09-3592 PMC1895780

[134] Hess DA , Bonde J , Craft TP , et al. Human progenitor cells rapidly mobilized by AMD3100 repopulate NOD/SCID mice with increased frequency in comparison to cells from the same donor mobilized by granulocyte colony stimulating factor. Biol Blood Marrow Transplant. 2007;13(4):398‐411. 17382247 10.1016/j.bbmt.2006.12.445 PMC1868544

[135] Kean LS , Sen S , Onabajo O , et al. Significant mobilization of both conventional and regulatory T cells with AMD3100. Blood. 2011;118(25):6580‐6590. 21989987 10.1182/blood-2011-06-359331 PMC3242720

[136] Lundqvist A , Smith AL , Takahashi Y , et al. Differences in the phenotype, cytokine gene expression profiles, and in vivo alloreactivity of T cells mobilized with Plerixafor compared with G‐CSF. J Immunol. 2013;191(12):6241‐6249. 24244025 10.4049/jimmunol.1301148 PMC3892143

[137] Devine SM , Vij R , Rettig M , et al. Rapid mobilization of functional donor hematopoietic cells without G‐CSF using AMD3100, an antagonist of the CXCR4/SDF‐1 interaction. Blood. 2008;112(4):990‐998. 18426988 10.1182/blood-2007-12-130179

[138] Chen YB , Le‐Rademacher J , Brazauskas R , et al. Plerixafor alone for the mobilization and transplantation of HLA‐matched sibling donor hematopoietic stem cells. Blood Adv. 2019;3(6):875‐883. 30890544 10.1182/bloodadvances.2018027599 PMC6436017

[139] Abraham M , Biyder K , Begin M , et al. Enhanced unique pattern of hematopoietic cell mobilization induced by the CXCR4 antagonist 4F‐benzoyl‐TN14003. Stem Cells (Dayton, Ohio). 2007;25(9):2158‐2166. 10.1634/stemcells.2007-0161 17525235

[140] Abraham M , Pereg Y , Bulvik B , et al. Single dose of the CXCR4 antagonist BL‐8040 induces rapid mobilization for the collection of human CD34(+) cells in healthy volunteers. Clin Cancer Res. 2017;23(22):6790‐6801. 28835380 10.1158/1078-0432.CCR-16-2919

[141] Abraham M , Beider K , Wald H , et al. The CXCR4 antagonist 4F‐benzoyl‐TN14003 stimulates the recovery of the bone marrow after transplantation. Leukemia. 2009;23(8):1378‐1388. 19322207 10.1038/leu.2009.56

[142] Peled A , Abraham M , Avivi I , et al. The high‐affinity CXCR4 antagonist BKT140 is safe and induces a robust mobilization of human CD34+ cells in patients with multiple myeloma. Clin Cancer Res. 2014;20(2):469‐479. 24246358 10.1158/1078-0432.CCR-13-1302

[143] Crees ZD , Stockerl‐Goldstein K , Vainstein A , Chen H , DiPersio JF . GENESIS: Phase III trial evaluating BL‐8040 + G‐CSF to mobilize hematopoietic cells for autologous transplant in myeloma. Future Oncol (London, England). 2019;15(31):3555‐3563. 10.2217/fon-2019-0380 PMC7421992 31495201

[144] Karpova D , Dauber K , Spohn G , et al. The novel CXCR4 antagonist POL5551 mobilizes hematopoietic stem and progenitor cells with greater efficiency than Plerixafor. Leukemia. 2013;27(12):2322‐2331. 24072044 10.1038/leu.2013.266 PMC3865534

[145] Karpova D , Bräuninger S , Wiercinska E , et al. Mobilization of hematopoietic stem cells with the novel CXCR4 antagonist POL6326 (balixafortide) in healthy volunteers‐results of a dose escalation trial. J Transl Med. 2017;15(1):2. 28049490 10.1186/s12967-016-1107-2 PMC5209880

[146] Tchernychev B , Ren Y , Sachdev P , et al. Discovery of a CXCR4 agonist pepducin that mobilizes bone marrow hematopoietic cells. Proc Natl Acad Sci USA. 2010;107(51):22255‐22259. 21139054 10.1073/pnas.1009633108 PMC3009824

[147] Srinivasan A , Panetta JC , Cross SJ , et al. Phase I study of the safety and pharmacokinetics of plerixafor in children undergoing a second allogeneic hematopoietic stem cell transplantation for relapsed or refractory leukemia. Biol Blood Marrow Transplant. 2014;20(8):1224‐1228. 24769325 10.1016/j.bbmt.2014.04.020 PMC4631218

[148] Konopleva M , Benton CB , Thall PF , et al. Leukemia cell mobilization with G‐CSF plus plerixafor during busulfan‐fludarabine conditioning for allogeneic stem cell transplantation. Bone Marrow Transplant. 2015;50(7):939‐946. 25867648 10.1038/bmt.2015.58 PMC4490031

[149] Michelis FV , Hedley DW , Malhotra S , et al. Mobilization of leukemic cells using plerixafor as part of a myeloablative preparative regimen for patients with acute myelogenous leukemia undergoing allografting: assessment of safety and tolerability. Biol Blood Marrow Transplant. 2019;25(6):1158‐1163. 30654137 10.1016/j.bbmt.2019.01.014

[150] Liu Q , Li Z , Gao JL , et al. CXCR4 antagonist AMD3100 redistributes leukocytes from primary immune organs to secondary immune organs, lung, and blood in mice. Eur J Immunol. 2015;45(6):1855‐1867. 25801950 10.1002/eji.201445245 PMC4461468

[151] Green MM , Chao N , Chhabra S , et al. Plerixafor (a CXCR4 antagonist) following myeloablative allogeneic hematopoietic stem cell transplantation enhances hematopoietic recovery. J Hematol Oncol. 2016;9(1):71. 27535663 10.1186/s13045-016-0301-2 PMC4989381

[152] Jin CH , Li Y , Xia J , et al. CXCR4 blockade improves leukemia eradication by allogeneic lymphocyte infusion. Am J Hematol. 2018;93(6):786‐793. 29603337 10.1002/ajh.25099 PMC5992083

